# New imaging modes for analyzing suspended ultra-thin membranes by double-tip scanning probe microscopy

**DOI:** 10.1038/s41598-020-60731-x

**Published:** 2020-03-16

**Authors:** Kenan Elibol, Stefan Hummel, Bernhard C. Bayer, Jannik C. Meyer

**Affiliations:** 1 Faculty of Physics, University of Vienna, Boltzmanngasse 5, A-1090 Vienna, Austria; 2 Institute of Materials Chemistry, Vienna University of Technology (TU Wien), Getreidemarkt 9/165, A-1060 Vienna, Austria; 3 Institute for Applied Physics, University of Tübingen, Auf der Morgenstelle 10, 72076 Tübingen, Germany

**Keywords:** Nanometrology, Scanning probe microscopy

## Abstract

Scanning probe microscopy (SPM) techniques are amongst the most important and versatile experimental methods in surface- and nanoscience. Although their measurement principles on rigid surfaces are well understood and steady progress on the instrumentation has been made, SPM imaging on suspended, flexible membranes remains difficult to interpret. Due to the interaction between the SPM tip and the flexible membrane, morphological changes caused by the tip can lead to deformations of the membrane during scanning and hence significantly influence measurement results. On the other hand, gaining control over such modifications can allow to explore unknown physical properties and functionalities of such membranes. Here, we demonstrate new types of measurements that become possible with two SPM instruments (atomic force microscopy, AFM, and scanning tunneling microscopy, STM) that are situated on opposite sides of a suspended two-dimensional (2D) material membrane and thus allow to bring both SPM tips arbitrarily close to each other. One of the probes is held stationary on one point of the membrane, within the scan area of the other probe, while the other probe is scanned. This way new imaging modes can be obtained by recording a signal on the stationary probe as a function of the position of the other tip. The first example, which we term electrical cross-talk imaging (ECT), shows the possibility of performing electrical measurements across the membrane, potentially in combination with control over the forces applied to the membrane. Using ECT, we measure the deformation of the 2D membrane around the indentation from the AFM tip. In the second example, which we term mechanical cross-talk imaging (MCT), we disentangle the mechanical influence of a scanning probe tip (e.g. AFM) on a freestanding membrane by means of independently recording the response of the opposing tip. In this way we are able to separate the tip-induced membrane deformation topography from the (material-dependent) force between the tip and the membrane. Overall, the results indicate that probing simultaneously both surfaces of ultra-thin membranes, such as suspended 2D materials, could provide novel insights into the electronic properties of the materials.

## Introduction

Scanning probe microscopes (SPMs) using small physical probes have become highly versatile tools for imaging and manipulation on rigid surfaces due to the many different types of interaction between the probe and the object that can be exploited. SPMs are typically able to map different types of forces, electric fields, currents, thermo power, and many other features of the sample, depending on the structure and materials of the tips and operation and feedback mode during the scan^1,2^. SPMs are also often used for nanoscale manipulation^3,4^, scanning probe lithography^5^ and electric contacting on very small areas^6^. Especially for the latter two purposes, multi-probe SPMs have become popular^7^. In this way, transport over very short length scales has been achieved^8–13^, however, the distance between tunneling tip and contact electrode (a large metal contact or another STM tip) could not be reduced down to the atomic scale due to the size of the probes and that both the tunneling tip and contact electrode are on the same side of the sample. Multi-probe SPMs with active parallel cantilevers have also been used for large scale imaging and lithography^14,15^. Moreover, the combination of low temperature STM and AFM on the same tip, which can measure the tunneling current and the force simultaneously, was shown to be beneficial for studying the graphite surface^16^.

SPM measurements on non-rigid surfaces, in particular suspended ultra-thin membranes, are more difficult to interpret because the forces between the probe and the membrane change the morphology of the membrane^17–21^. For a very thin membrane, the membrane follows the tip, rather than vice versa. In that case, the membrane is deflected as far as possible under the attractive or repulsive force of the tip, and the scan profile traces the membrane deflection that is caused by the tip force at each point^18^. Recently, it was demonstrated that ultra-thin membranes (in that case made from few-layer graphene) can be accessed with two independent scanning probes from opposite sides of the membrane^22^, and that a mechanical deformation can be induced with one probe while being observed with the second probe.

Now, we demonstrate new imaging modes that become possible by using signals from two tips on opposite sides of the membrane. In general, one of the probes is scanned across the suspended membrane as usually done for imaging, while the second probe is held stationary in the (x,y) plane of the membrane with its height (z) feedback on or off. Signals from the stationary probe, such as its z position, or electric current, are fed into the external input of the actively scanning probe and are thereby mapped as a function of the scanning probe’s position. In this way, a large number of new imaging modes become conceivable - in principle, any scan mode of the moving probe could be combined with any signal from the stationary probe, with the exception of combinations that would lead to a collision of the probes.

## Experimental Setup

**Figure 1:**
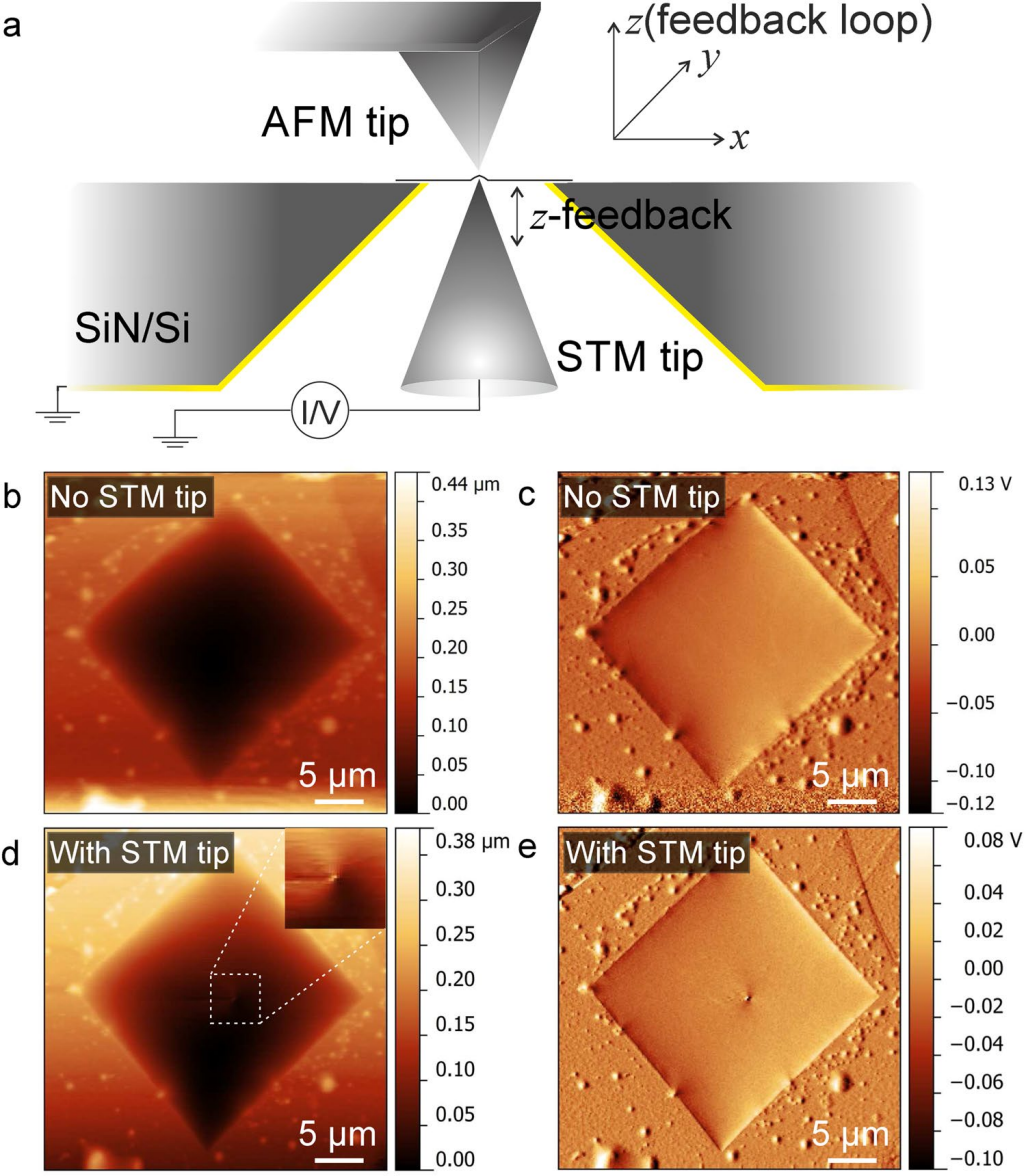
(**a**) Schematic illustration of the setup. (**b**) AFM topography and (**c**) error signal images of a few-layer graphene membrane. (**d**) AFM topography and (**e**) error signal images of the same sample while a deformation is being induced by a stationary STM tip (with z feedback loop on). Inset in panel d is the close-up image of the area in white dashed frame.

The setup consists of a scanning tunneling microscope (STM) and an atomic force microscope (AFM) arranged on opposite sides of the suspended membrane (Fig. 1a, see also Supplementary information). Both devices are tip-scanning with the sample remaining stationary, which is an important prerequisite for the dual-probe measurements as described here. The sample carrier is a silicon chip with a thickness of 200*μ*m, into which a pyramid shaped hole is etched by anisotropic etching from one side of the chip (special made by Silson inc., UK). The result is an approximately square shape aperture with a size of 5 − 20*μ*m on the top surface of the chip, which faces the AFM scanner, and onto which the membranes are placed (Fig. 1a, Supplementary Figs. 1,2). The back side of the chip is coated with 5nm of chromium and 20nm of gold for electrical conductivity, prior to placing the 2D material membrane.

A coarse alignment of the scanners and the sample is done with the help of an optical microscope above the AFM scanner. In addition, the inverse pyramid shape of the sample carrier geometry helps to guide the STM tip towards the free-standing membrane. The STM is approached into the pyramid shaped hole on the back side of the chip, and can be navigated into the hole until it reaches the membrane from the other side. For this purpose, the STM is approached into the pyramid hole and a very small scan is used to detect whether the tip has landed on the side surface or on the membrane. Then, a coarse displacement is used to move to the center of the pyramid. After a few steps, the membrane is reached. An STM scan showing the membrane and two side walls of the pyramid is shown in Fig. 3b and Supplementary Fig. 3b.

**Figure 2:**
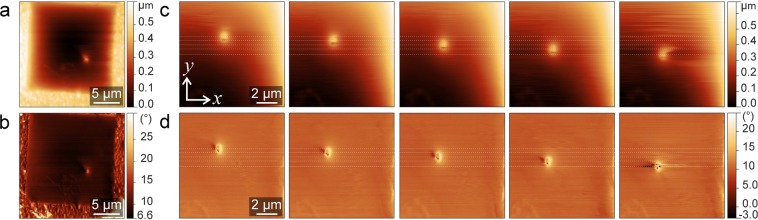
Large-scale and close-up (**a**,**c**) topography and (**b**,**d**) phase images, respectively, of a multilayer graphene membrane with an STM tip apprached on the opposite side. The STM tip is moved in -*y* direction before each AFM scan in (c,d). Tunneling parameters for the STM tip are U = 0.4 V and I = 0.2 nA. Damping setpoint of the AFM is 20%.

**Figure 3:**
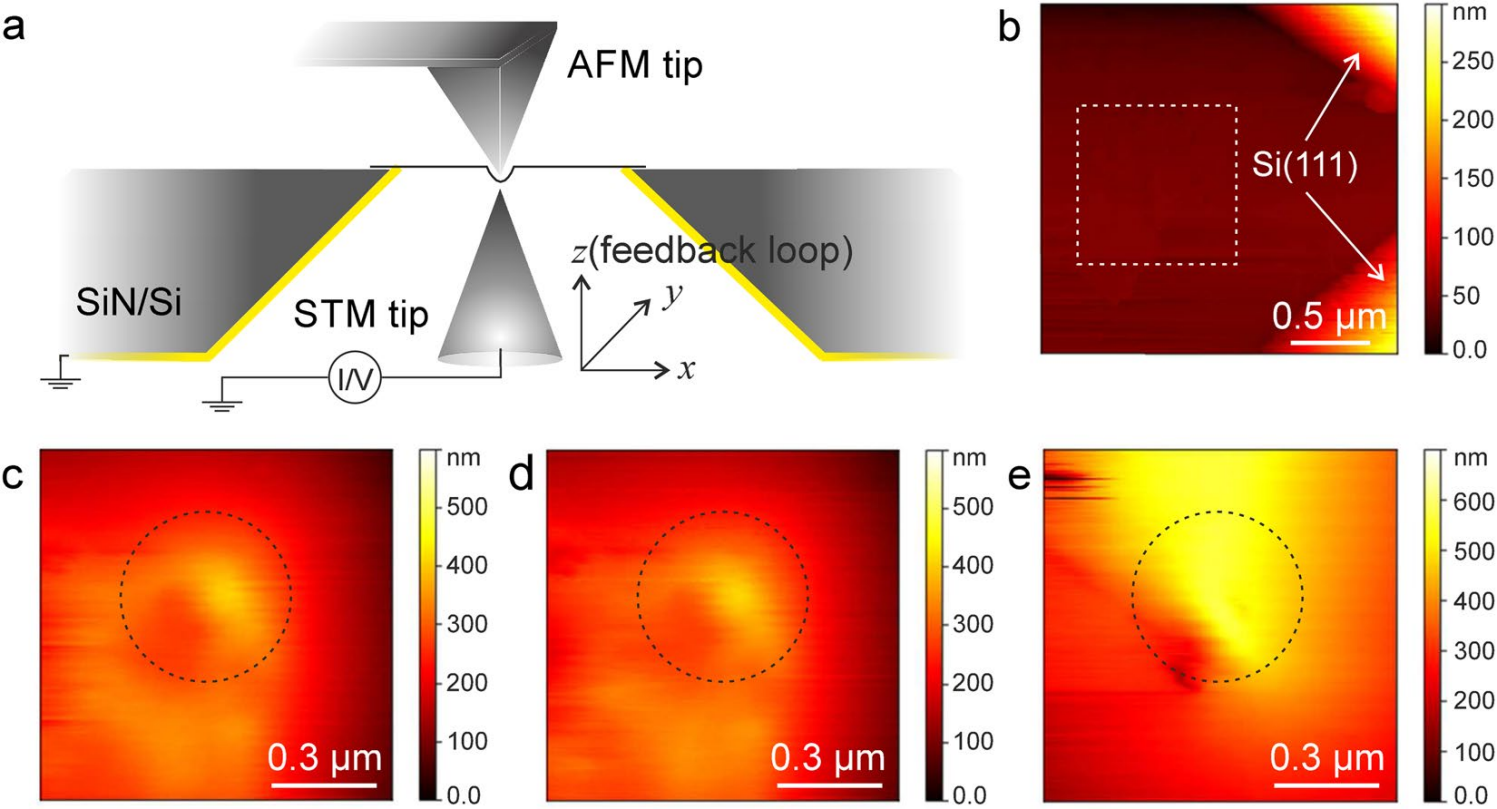
(**a**) Schematic of the experiment. (**b**) STM topography image of a multilayer MoS_2_ membrane. (**c**–**e**) STM topography images of the area in white dashed frame on panel b while the membrane is deformed by an AFM tip with the force set points of 0.2 nN, 0.4 nN and 0.6 nN, respectively. The position of AFM tip is pointed by dashed circles. Tunneling parameters for the STM tip are U = 0.4 V, I = 0.5 nA.

The suspended membranes used in this work are few-layer samples of 2D materials, either few-layer graphene or MoS_2_. They were prepared by mechanical exfoliation on a silicon substrate with a 90 nm oxide layer and subsequently transferred onto the target substrate with the aperture. Based on the optical characterization, the graphene flakes used in the experiments comprise of 5–15 layers of graphene^23,24^, and the MoS_2_ membranes in Fig. 3 and Fig. 11 comprise ca. 30 layers.

Once the tips are within each other’s scan range, one of the tips (usually the STM) is held stationary on the membrane, and the deformation induced in the membrane can be found by the other tip. An example of AFM (tapping mode) overview image with the STM tip approached on the other side of the membrane is shown in Fig. 1. In this case, the STM z-position feedback loop is active during the AFM scan. Nevertheless, the STM tip position can be recognized as a distortion in the apparent shape of the membrane. By zooming in to the other tip’s position, the two tips can be precisely aligned.

Unfortunately, our setup is not gentle enough to enable measurements on thinner (ideally single-layer) samples. Although we were able to prepare suspended mono-layer graphene membranes on these carriers, they were immediately destroyed when approaching one of the tips. Even with thin multi-layer membranes, the experiments were still severely limited by the frequent breakage of membranes. We attribute this to problems with the specific scanners or their control electronics, since the breakage occurred already with only one tip, and since we were able to successfully scan mono-layer samples in other types of AFMs and STMs repeatedly without breakage. Also, STM scans of suspended single layer graphene can be found in the literature, usually in ultra-high vacuum (UHV)^18–20,25,26^ but also in ambient conditions^27^. This indicates that experiments similar to those demonstrated here should also be possible with single-layer samples, with an improved setup and control electronics, and possibly UHV conditions.

 Figure 2(c,d) shows the close up of the AFM scan on the STM tip position. Here, the AFM amplitude and phase are shown. The change in the phase indicates that the sample surface is more rigid where the other tip is. In the image sequence, the STM tip is displaced in -y direction, and so does its apparent position as observed from the AFM side. This confirms that the tip does not induce a permanent deformation in the membrane. It is also possible to keep the AFM tip stationary, and image the membrane using the STM tip. In Fig. 3, the AFM was switched to contact mode and the tip was kept stationary on the membrane (with z feedback loop on, ie., maintaining a constant force). The STM images show the location of the AFM tip and the apparent deformation in the membrane.

**Figure 4:**
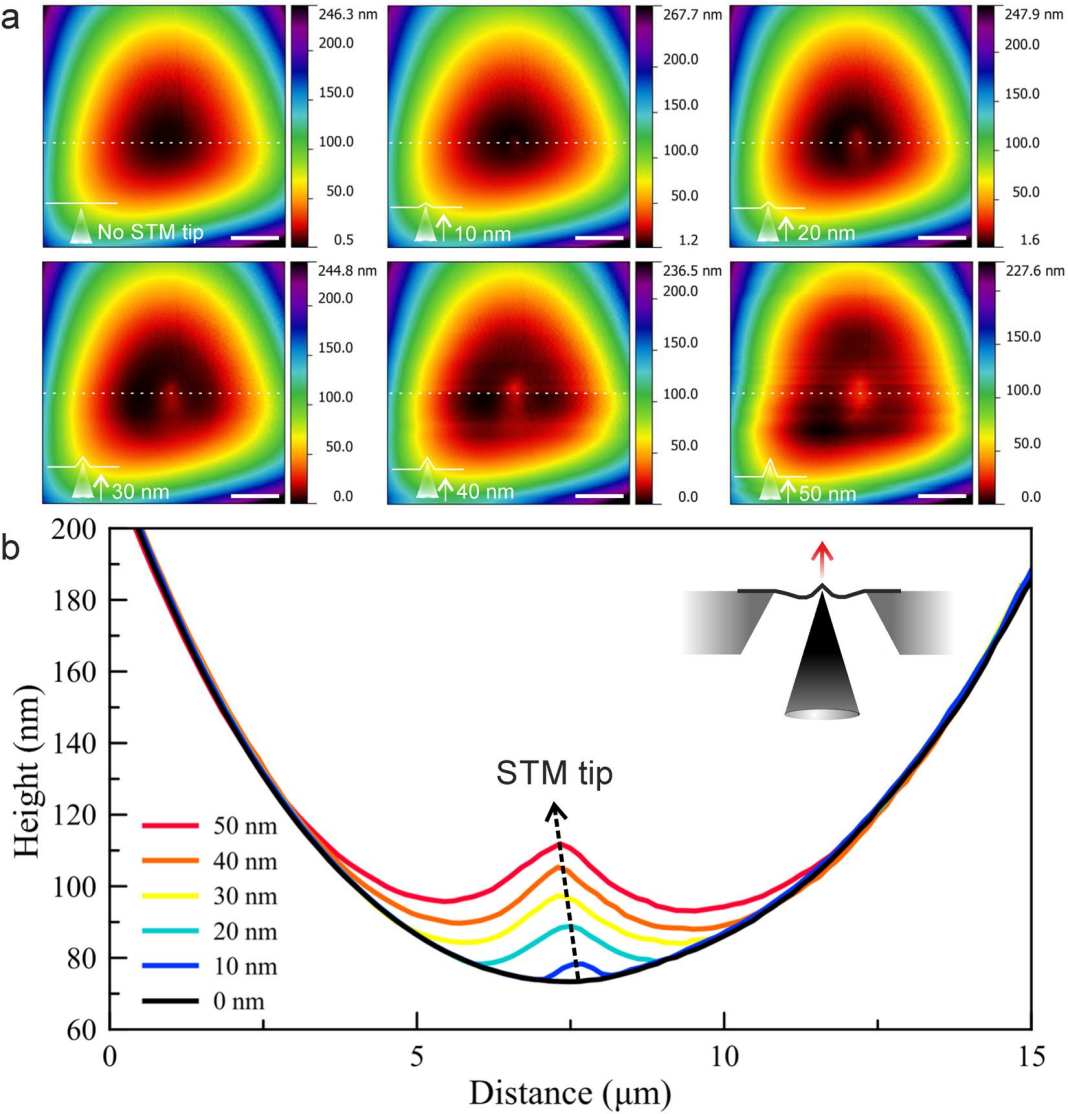
(**a**) Series of the contact mode AFM topography images for different STM *z*-piezo positions. STM feedback is off. (**b**) Line profiles along the white dashed lines on the topography images. Inset shows the schematic illustration of the deformed membrane. The scale bars are 3 *μ*m.

The first images of a sample on which several measurements were performed are shown in Fig. 4 (further measurements on the same sample are shown in Figs. 6 and 7). Here, the AFM operates in contact mode with a force set point of 2 nN. The STM is placed close to the membrane, and its feedback loop is switched off. The measurement is repeated with different z displacement of the STM tip as indicated. Indeed, the topography AFM images in Fig. 4 suggest a deformation is induced in the suspended membrane, and its height depends on the z position of the STM tip. However, the electrical measurements as described in the following section reveal that this is a dynamic effect of the membrane changing its position during the scan.

**Figure 5:**
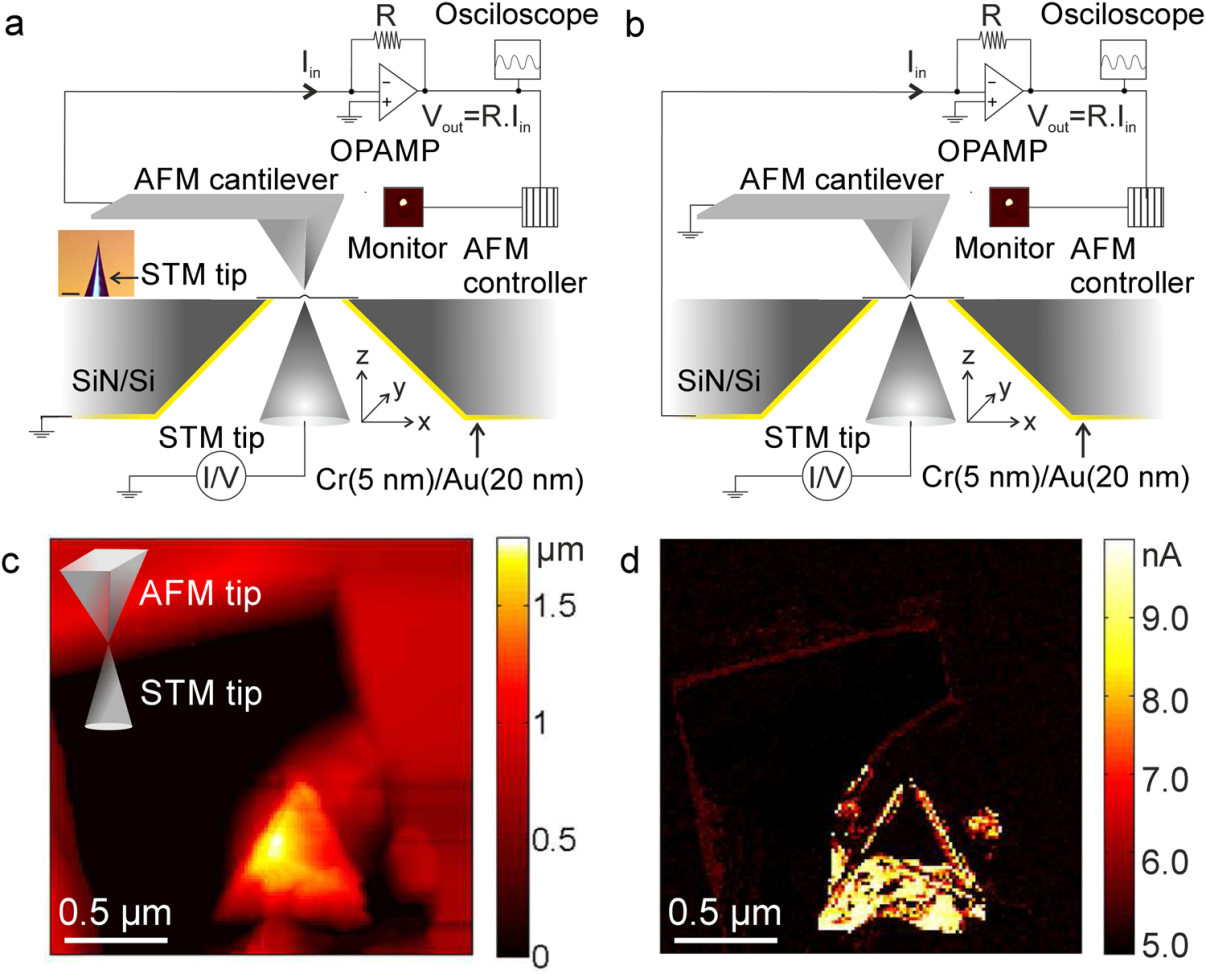
Schematic illustration of the ECT imaging technique enabling to record signal (**a**) by a conductive AFM cantilever or (**b**) directly from the sample. An optical microscope image of a tungsten (W) STM tip is shown in the inset of panel a. The scale bar on the optical micrograph is 20 *μ*m. (**c**) AFM topography image and (**d**) simultaneously recorded ECT image when STM tip is stationary (with z feedback loop off), and no membrane is present. Here the signal is recorded using the method shown in panel a. Inset shows the schematics of AFM and STM tips interacting. The number of pixels is 256 × 256.

**Figure 6:**
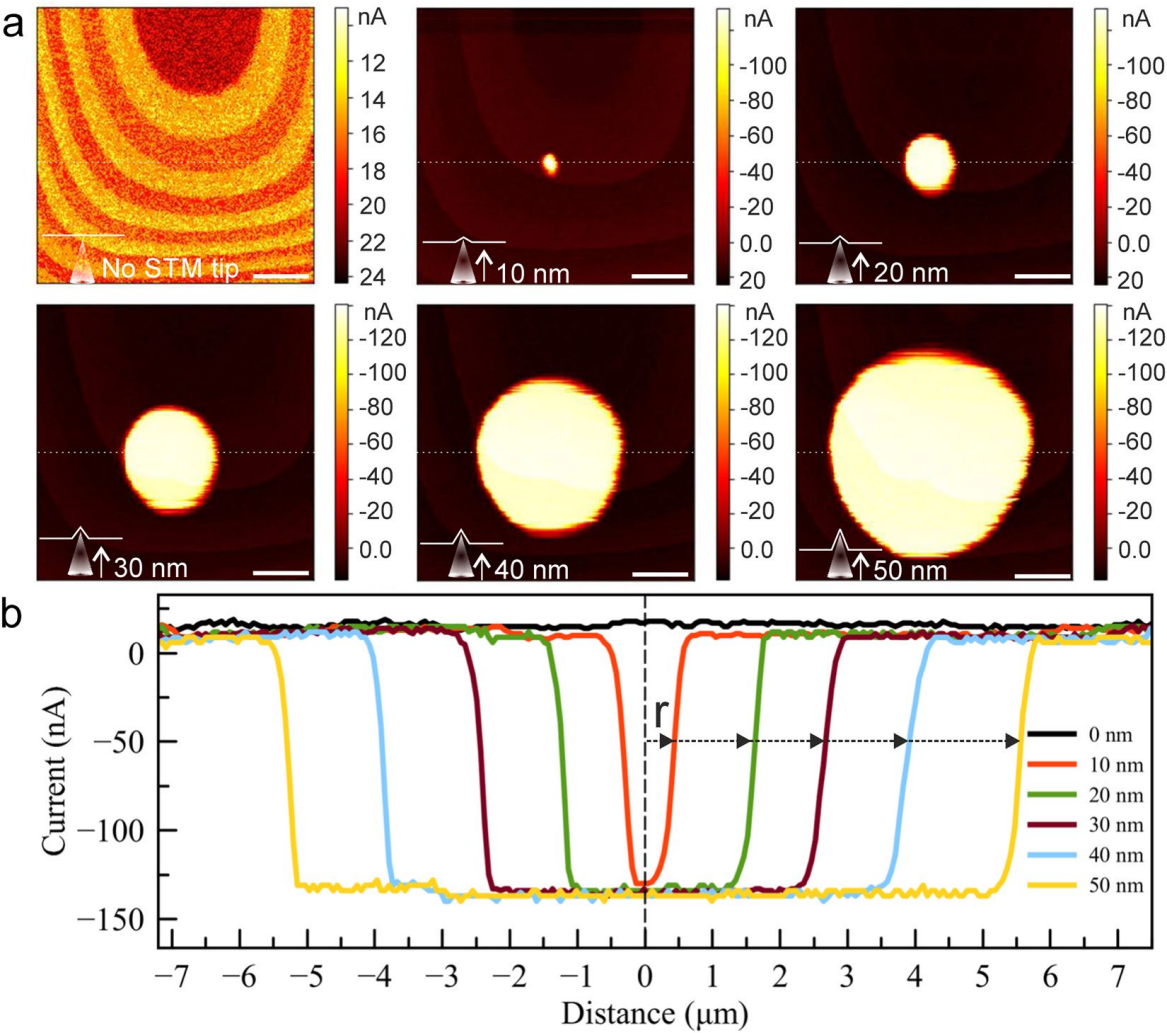
(**a**) Series of constant height ECT images recorded at different z positions of the STM tip (feedback off). The scale bars are 3 *μ*m. (**b**) Line profiles recorded along the white dashed lines on the images in panel a. The arrows indicate the radius(r) of currents.

**Figure 7:**
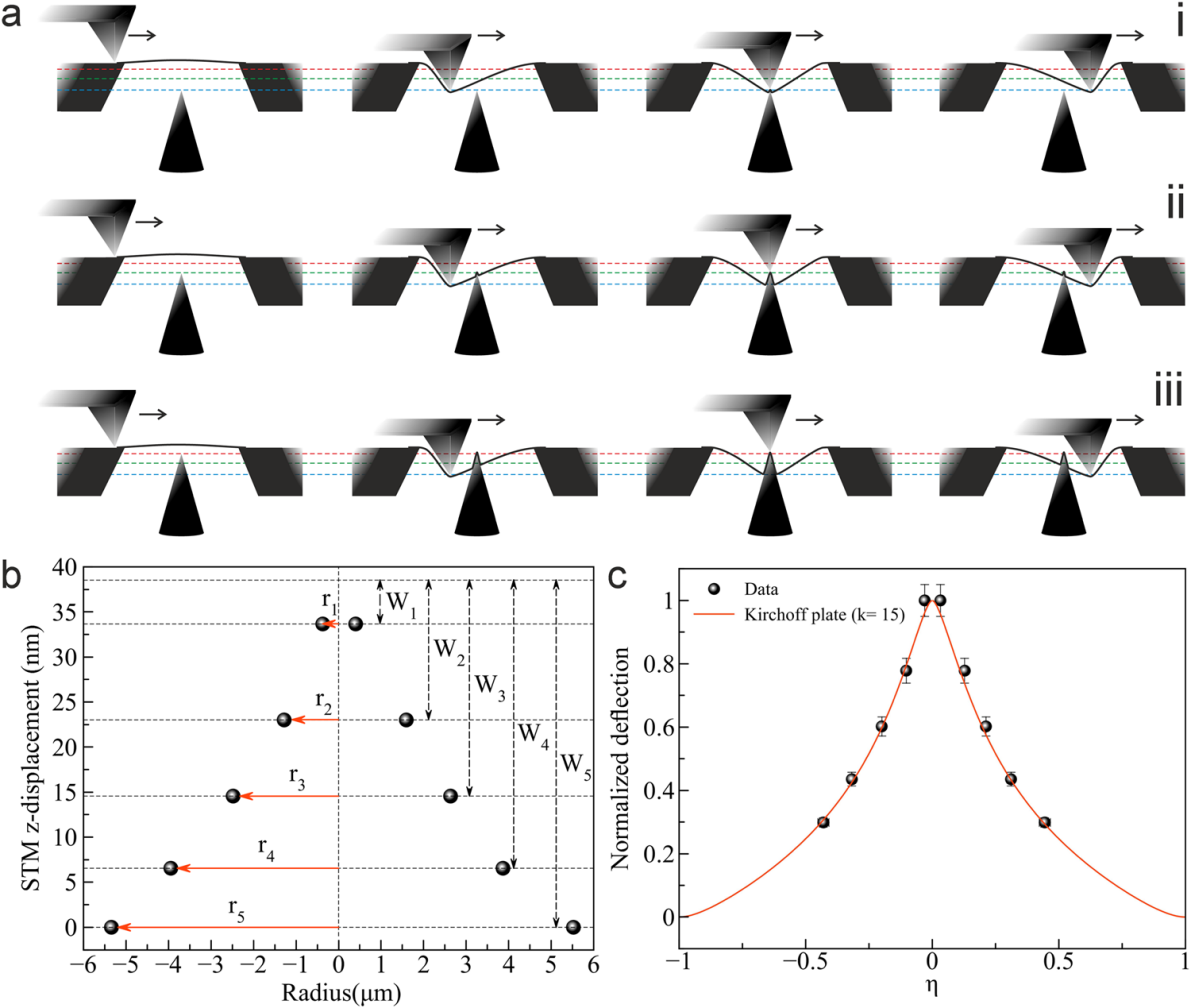
(**a**) Schematic illustrations showing the interaction between SPM tips and membrane for different z positions of the STM tip. Here the STM tip is at different heights (firstly at blue line (i), secondly at green line (ii) and lastly at red line (iii)). (**b**) STM z-displacement as a function of radius of the signal received. The radii (r) were recorded where the current is -50 nA in Fig. 6b, whereas the STM z-displacements (W) were measured from Fig. 4b. (**c**) Calculated deflection fitted to the experimental data in panel b versus normalized radial distance *η*. The membrane’s radius a is 12.5 *μ*m.

## Electrical cross-talk imaging

We begin with the first example of a new imaging mode that becomes possible with two probes and which we term “electrical cross-talk imaging” (ECT). In this case, a current is recorded between either of the tips and the sample (or between the two tips), as a function of the position of the scanning tip. One of the ideas behind such measurements is that, in principle, the distance between the two tips can be as small as the thickness of the membrane and then a transport measurement could be done over this short distance. Although we could not collect extensive transport data (as should be possible by the variation of voltage and current as well as different forces or deflections of the membrane) due to breakage of membranes, the possibility to position tips across the membrane and measure electric signals nevertheless indicates that this should be possible with a revised setup.

 Fig. 5a,b shows the schematic of the two electrical modes. In both cases, the STM tip is kept at a given bias voltage. The z feedback of the STM tip is off (constant height mode) for the measurements described first. In Fig. 5a, the current is measured with a conducting AFM tip, and the sample carrier is connected to the ground. In this case, the current recorded on the AFM tip directly flows across the membrane. In Figure 5b, the current is measured from the sample carrier, while the AFM tip is grounded or may also be a non-conducting tip. In this case, the contact between the STM tip and the membrane is modulated by the AFM tip, which could be useful to apply strain to the membrane.

**Figure 8:**
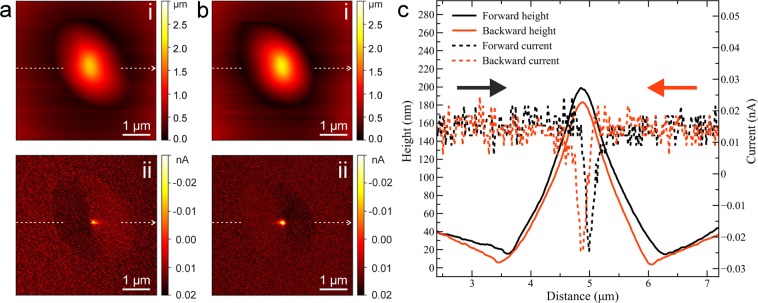
(**a**,**b**) Simultaneous (i) topography and (ii) ECT images of a multilayer graphene membrane in the forward and backward scan directions, respectively. (**c**) Line profiles recorded along the white dashed lines on the forward and backward topography and ECT images. The damping setpoint of the AFM cantilever and number of pixels are 20% and 256 × 256, respectively.

Figure 5c shows a measurement without membrane, i.e., the AFM is scanning over the hole in the substrate with the STM tip approached into the hole from the other side. The current is recorded from the AFM tip. In the topography, the STM tip appears with a triangular pyramid shape, which is in fact the shape of the AFM tip and appears due to the tip convolution of the AFM tip with the much sharper STM tip. The square shaped aperture in the substrate is visible at the edges of the image. The current image (Fig. 5d) does not show the substrate and the aperture. Instead, it only shows points in the scan where an electric connection between the AFM and STM tip is obtained. While the triangular shaped pyramid of the AFM tip can be discerned, it appears that there are regions on the AFM tip where no current can flow. We attribute this to the AFM tip being not perfectly coated by the metal layer, as there are some regions within the boundary of the tip where no current is recorded. This might also be due to a peeling or oxidation of metal coating during AFM scanning. We therefore continue the next experiment with the second option shown in panel b, i.e., current recorded between the STM tip and the sample ground, as a function of the AFM tip position.

Figure 6  shows the current recorded between sample and ground, with the STM tip placed at different distances behind a few-layer graphene membrane. This data was recorded simultaneously with the topography that was shown already in Fig. 4. In the first image of Fig. 6a, the STM tip is far away from the membrane, and the current that flows between the sample and the ground is very small (the origin of the weak ring shaped current pattern unfortunately remains unclear to us). However, if the STM tip is moved closer to the membrane, a circular region with a current that corresponds to the limiting current of the STM tip’s power supply (ca. 140 nA) appears. The diameter of this region grows as the STM tip is moved closer to the membrane.

The explanation of this observation is straightforward: The AFM tip, scanning in contact mode, pushes the membrane towards the STM tip, and the maximum possible current flows as soon as a contact is obtained. This also means that the STM tip is not in contact with the membrane when the AFM tip is laterally far away from it. Hence, the shape of the suspended membrane as seen in Fig. 4 is not a static configuration, but rather, shows the profile of how the membrane is dynamically deformed by the AFM tip. The current flows whenever the deflected membrane touches the STM tip, which happens when the AFM is close to the STM tip position. This process is illustrated in Fig. 7a. The STM z-displacement as function of radius of the current is shown in Fig. 7b.

The membrane comes into contact with the STM tip when the deflection of the membrane surrounding the AFM indentation reaches the height that is given by the z position of the STM. Hence, with several measurements at different z positions of the STM, we have obtained a profile of the membrane deflection surrounding the AFM tip. The experimental data is compared to the Kirchoff plate theory^28^, which describes the deflection of a membrane under a point load. For this purpose, the data is shown in normalized coordinates in Fig. 7c. Here, the normalized radius *η* = (r/a), where r is the radius and a the diameter of the suspended area. The deflection is normalized so that the maximum deflection (normalized deflection of 1) is at the center, and linearly rescaled so that extrapolation with the fitted Kirchhoff plate model arrives at zero at the boundary. The data acquired by the ECT measurement fits well to a membrane model with an in-plane tension parameter of k = 15 (see Supplementary Fig. 4).

The ECT experiment can also be done with the AFM in tapping mode. The topography and ECT images of a multilayer graphene membrane are shown in Fig. 8. The localized electrical signal appearing on the ECT images is less than the maximum value of the short circuit current. We assume that this is due to the tapping mode AFM operation, where the membrane might be in contact with the STM tip only during a small fraction of the time during each oscillation period, and we can only measure a current average. The line profiles taken along the topography and ECT images recorded for forward and backward scan directions are shown in Fig. 8c. They show that an almost point-like spike in the current is received when the AFM tip moves across the STM tip. In particular, there is a difference between the forward and backward trace, which indicates that the membrane is pushed into contact with the tip by a slightly too slow feedback loop.

**Figure 9:**
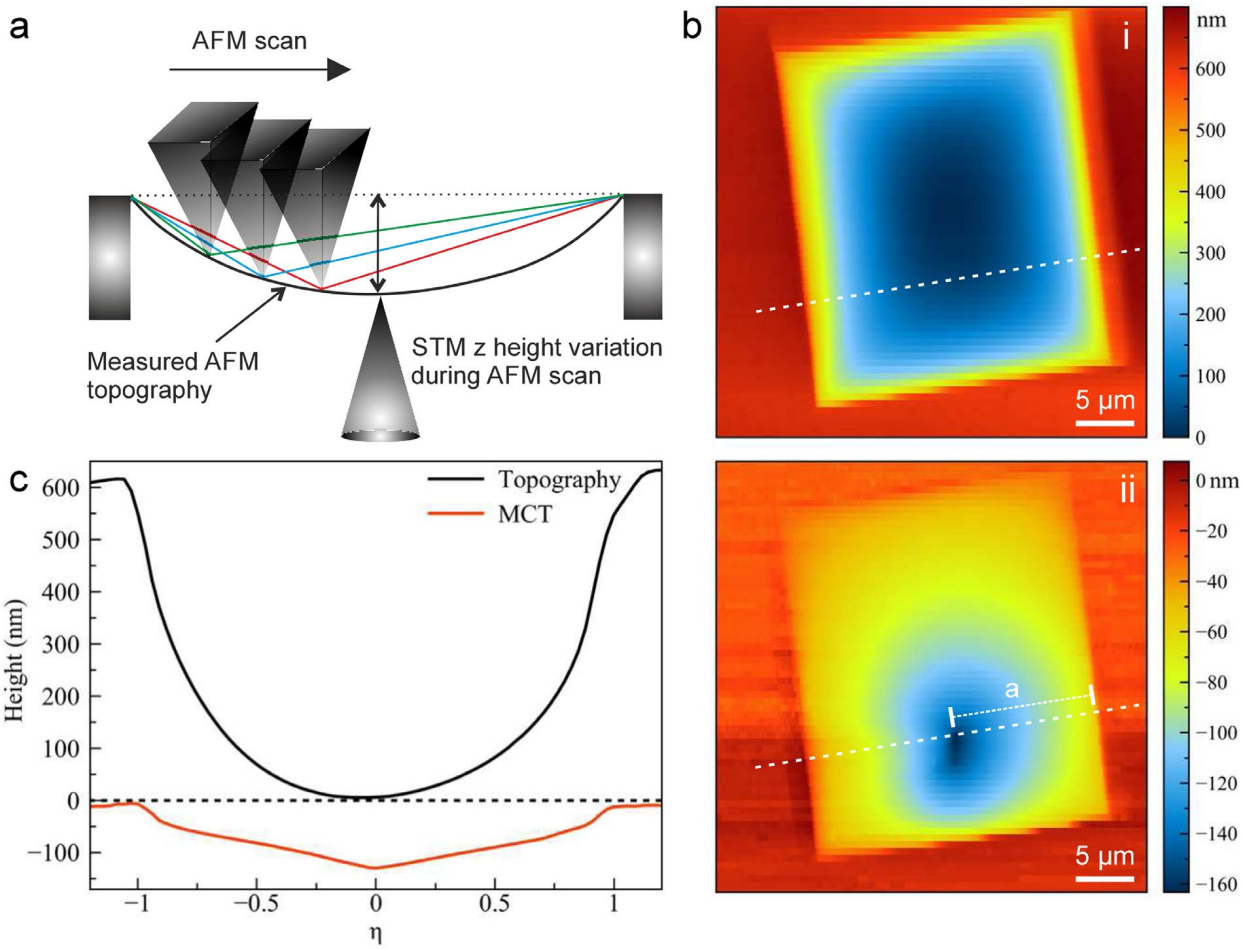
(**a**) Schematic of a membrane deformed by the AFM tip, here assumed to be pushing down on the membrane. The black line indicates the AFM trace, while the green, blue and red lines indicate actual membrane configurations at each point of the scan. The STM is kept stationary in (x,y), while its z feedback is on. (**b**) AFM topography in contact mode (i) and Mechanical cross talk (MCT) (ii) image, showing the z position of the STM tip as a function of the AFM’s (x,y) position. Tunneling parameters for the STM tip are U = 0.4 V and I = 4 nA. Force set point of the AFM is 2 nN. (**c**) Height profiles recorded along the white dashed lines on panel b(i–ii).

## Mechanical cross-talk imaging

We now move on to describe a mode that we termed “mechanical cross-talk” (MCT) imaging, where the z piezo feedback of the stationary tip (in our case, the STM) is active, and the z piezo position is recorded as a function of the other tip’s x,y position. In this mode, STM tip’s x,y position is kept constant while AFM tip is approaching or scanning. Fig. 1a can serve to illustrate this mode. We point out that the AFM topography, error signal and phase images as presented already in Figs. 1,2 were also recorded with the STM z feedback active. But now, we feed the STM’s z piezo signal as external input into the AFM scanner, which allows us to map it as a function of the AFM’s x,y position.

 Figure 9 shows a first example of this mode, with the AFM operating in contact mode. The sample is a ca. 15-layer graphene membrane. The contact mode AFM measurement on the relatively thick membrane does not reveal the STM tip position in the topography (Fig. 9b,i). The MCT image (Fig. 9b,ii) reveals again that the membrane is dynamically deformed during the AFM scan.

**Figure 10:**
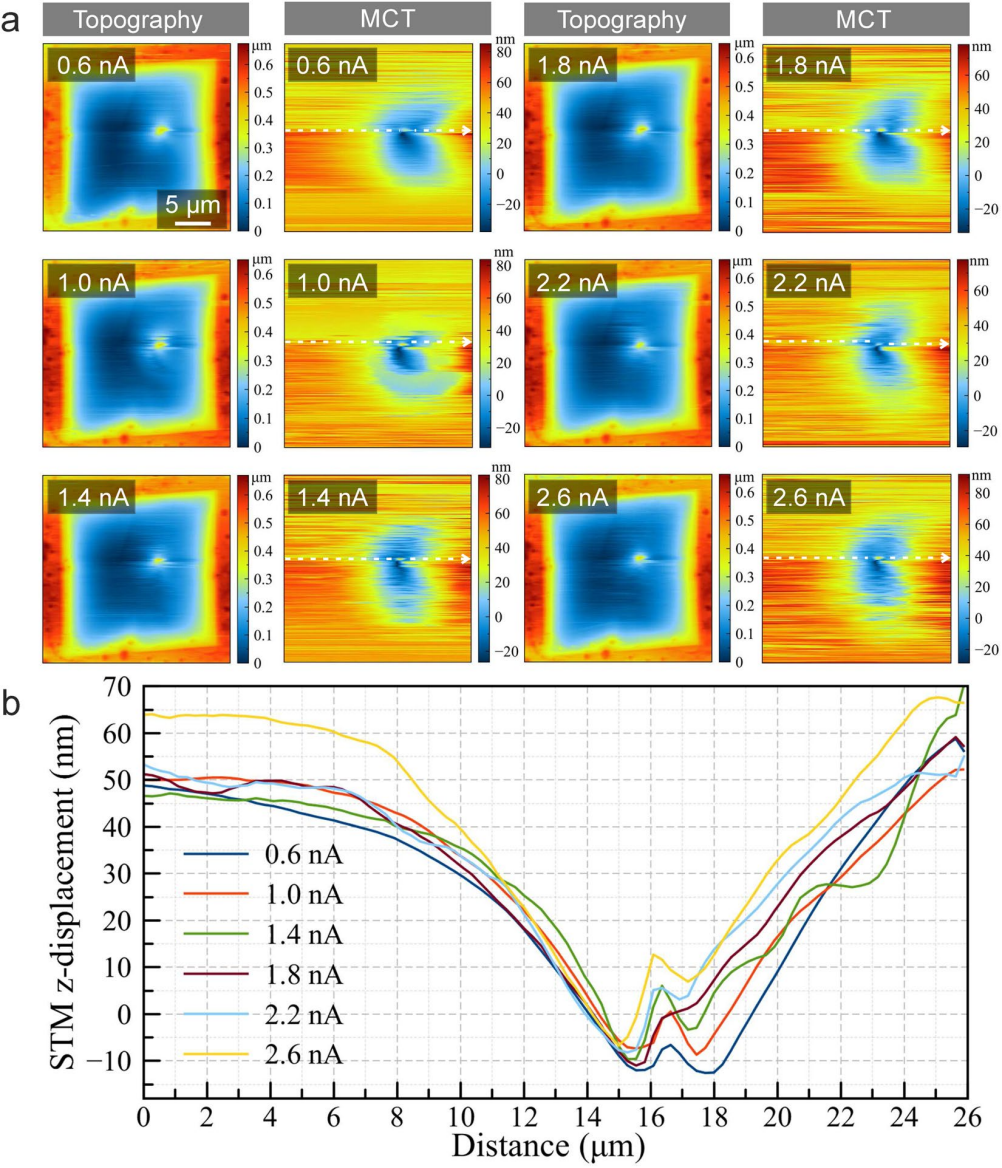
(**a**) Tapping mode AFM topography (setpoint has been 80% of the free-oscillation) and MCT images of a few-layer graphene membrane at different STM tunneling parameters (U= 0.4 V for each frame). (**b**) Profile plots of the MCT images.

We reiterate that the STM tip is held in contact with the membrane via its feedback loop. Hence, its z position will reveal the local position of the membrane, as a function of the other tip’s position (however, it would not be able to follow the ca. 90kHz oscillation of the AFM tip in tapping mode). In the cross-talk image (Fig. 9b,i), the height of the STM is practically constant (except for noise) in the periphery of the image, outside of the aperture where the AFM tip is on the support frame. However, one can see that the membrane is pushed downwards, towards the STM tip, as soon as the AFM tip is above the free-standing part of the membrane. Correspondingly, the profile plot (Fig. 9c) shows a clear step at the edge of the membrane. The maximum membrane deflection as seen by the STM tip is when both tips are aligned, at the center of the profile plot in Fig. 9c.

We now move on to MCT measurements with the AFM in tapping mode. The advantage of the tapping mode is that the interaction between the AFM and the membrane is weaker than in contact mode, due to significantly smaller shear forces, thereby enabling measurements on thinner membranes with a lower risk of breakage. However, the interaction especially in presence of the other tip is much more complex, and not yet fully understood. Fig. 10a shows topography and MCT images of a few-layer graphene membrane. In this case, the position of the STM tip can be seen in the topography images, indicating that the membrane shape is not dominated by the AFM, but rather controlled by the complex interaction of both tips.

**Figure 11:**
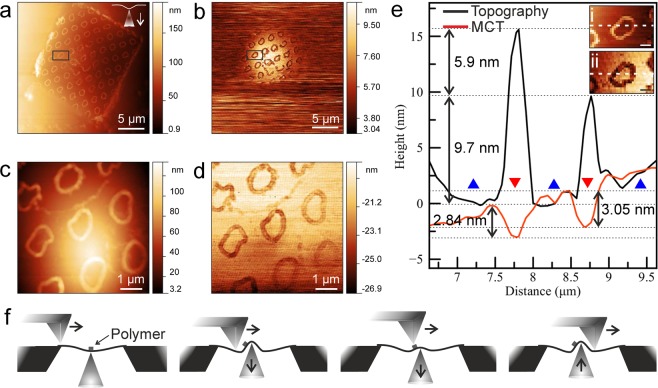
(**a**) Topography and (**b**) MCT images of a multilayer MoS_2_ membrane. Tunneling parameters for the STM tip are U = 1.0 V and I = 0.2 nA. (**c**,**d**) Close-up topography and MCT images recorded on the same sample. (**e**) Height profiles recorded along the white dashed lines on the images shown in the inset. Inset shows the close up (i) topography and (ii) MCT images taken from the dashed frames in panels a and b. The blue (up) and the red (down) arrows show the direction of the STM tip movement. The scale bars on the images in inset are 0.5 *μ*m. (**f**) Schematic illustration of the interacting SPM tips and sample. The direction of STM and AFM tips’ movement is shown by black arrows.

The MCT signal in Fig. 10a is constant (except for noise) in the periphery of the image, and unlike the previous example, does not reveal the edges of the suspended area of the membrane. Hence, the position of the STM tip, and thus the position of the membrane at its location, does not change significantly when the tips are at a lateral distance of more than ca. 5 *μ*m (the size of the suspended membrane is ca. 20 *μ*m). However, the membrane is moving downwards (towards the STM) when the AFM tip comes within this radius of the STM. This shows that the membrane is not strongly pushed down by the AFM tip, like it was the case in contact mode.

A so far unexplained observation is that the STM tip moves upwards again when the tips become aligned or are very close to each other (<0.5*μ*m laterally, central spike in the topography and MCT data in Fig. 10a,b). This behaviour was observed several times in very thin (5–10 layer) few layer graphene membranes. It can not be explained by assuming that both tips push or pull on the membrane with a constant force, in which case one would expect the cross-talk image to show deflection to the same side throughout the image. Since the strength of this feature weakly depends on the tunneling current (Fig. 10b), a potential explanation is that the feedback of the STM cannot follow the fast tapping interaction of the AFM tip with the membrane and hence, overshoots when the AFM tip is in close proximity of the STM interaction area and passes the STM tip. It is also conceivable that the density of states (DOS) of the membrane is affected by the presence of the AFM tip, e.g. by the localized strain, which would affect the tunneling probability and hence cause a response in the feedback loop. Further experiments would be needed in order to clarify the reason for this observation.

The next example (Fig. 11) shows again an MCT measurement with the AFM in tapping mode, but now on a ca. 20 nm MoS_2_ membrane that has a pattern of contamination on the surface as a result of the transfer procedure. The transfer of the membrane onto the chip was done using a Quantifoil(TM) TEM grid made from plastic, which can be dissolved but it leaves some periodic, ring-shaped contamination on the MoS_2_ (also visible in a TEM image of the membrane, shown in the Supplementary Fig. 2c). Due to the sample preparation process, this contamination pattern must be on the AFM side of the sample.

In the scanned AFM topography image (Fig. 11a), we can see the edges of the aperture over which the membrane was placed, the slightly non-flat membrane, and a weak upward bulge of the membrane at the position of the STM tip (confirmed by moving the STM tip sideways). The feature of the tip is less sharp than in the previous example, presumably due to the much thicker membrane. In the MCT image (Fig. 11b), we see a signal corresponding to that the STM tip is extended whenever the AFM tip comes closer than ca. 5 *μ*m. Hence, we can conclude that in this case the membrane is pulled upward by the AFM (i.e., towards the AFM), and this displacement is detected by the STM and mapped in the MCT image.

Most strikingly, we can see the contamination pattern in the AFM topography image on all of the free-standing membrane, and in particular also in the cross-talk image within the area where a cross talk signal is obtained. Assuming again that the STM tip follows the position of the membrane, this means that the height of the membrane (at the position of the STM) depends on whether the AFM tip is above a polymer contamination or above the cleaner MoS_2_ regions. Since the contamination is on the AFM side, and since the STM tip does not scan, this implies that the force between the AFM and the membrane depends on the local presence of the contamination: The membrane is pulled towards the AFM more strongly where the MoS_2_ has less contamination. This varying force between the AFM and the membrane leads to a displacement of the membrane, which is then detected by the STM. The features are most clearly visible at a lower STM current (Supplementary Fig. 5). This can be understood by considering that at lower current, the STM tip applies less force to the membrane and thus mostly acts as a position sensor, while at higher current the STM tip applies a stronger force to the membrane so that its position is less influenced by small forces acting elsewhere on the membrane.

## Discussion

Our experiments indicate a variety of new measurements that become possible with two probes located on opposite sides of an ultra-thin membrane. First of all, it is possible to align the two probes using the deformation of the membrane as caused by a stationary probe as reference, and locating it with the second (scanning) probe. This was shown previously with two STM scanners (Ref. ^22^) and is extended here also for a combination of AFM and STM, with different imaging modes (tapping mode and contact mode AFM).

Generally, when used as mechanical probes, the use of multiple probes makes it easier to disentangle the tip-membrane interaction forces from deformations of the membrane. In the present work, the electric signals between the tips and the membrane were also used to gain additional information on the membrane shape. In principle, it should also be possible to measure electronic properties of the membranes on very short length scales. In previous multi-tip SPM measurements of 2D materials^11,29,30^, the samples were not free-standing and the distance between electrodes could not be reduced down to the atomic scale. We expect that two probes on opposite sides of a one-atom or few-atom thick membrane may ultimately enable transport measurements at very short length scales. When both probes are vertically aligned on opposite sides of a thin membrane, the transport is expected to be ballistic along vertical direction due to the mean free path of electrons which is much longer than the distance between probes. With the increase of probe separation, the transport might be diffusive in lateral direction if mean free path is shorter than the spacing between tips^31^. A detailed theoretical analysis of the intriguing new measurements that can become possible with two STM tips and thus transport across very short distances, especially in presence of impurities, can be found in refs. ^32–34^. Double-tip experiments might also enable probing the strain induced shifts in the band gaps and piezoresistivity in 2D materials’ membranes^35^, or studying the strain induced magnetic properties of 2D van der Waals magnetic semiconductors by using specialized tips^36^.

Both the ECT and MCT experiments confirm that the observed trace in an AFM scan of a suspended membrane does not show the static configuration of the membrane (as it would be without the probe), but rather, a trace of how far the membrane can be displaced at each point. A membrane can be pulled by long range electrostatic forces or pushed by short range repulsive forces acting between the tip and the sample^20,25,27,37–39^, depending in the case of STM on the tunneling parameters, and in the case of the AFM on the force setpoint (contact mode) or damping setpoint (tapping mode). During an AFM or STM scan, these forces deflect the membrane as far as it is possible without the introduction of significant in-plane strain (for the estimate of strain see supplementary information). Hence, the resulting scan profiles and images show how far the membrane can be pushed or pulled at each point.

By placing the STM tip into a fixed position close to the membrane (Fig. 6) and recording the electrical signal, we have built a nanoscale switch that provides a current whenever the membrane comes into contact with the STM tip. This happens during the AFM scan, when the membrane is pushed towards the STM tip. In Figs. 6,7, we have shown such a measurement with the STM tip at different distances from the membrane. If we now assume that the deformation of the membrane around the AFM tip moves along with the AFM tip during the scan, the height vs. radius plot directly shows how the membrane is deformed under the AFM tip (Fig. 7b). This assumption will be most valid close to the AFM tip, where the membrane shape should be dominated by the indenting force of the AFM. The result is the first direct measurement of a 2D material membrane shape under indentation from an AFM tip.

**Figure 12:**
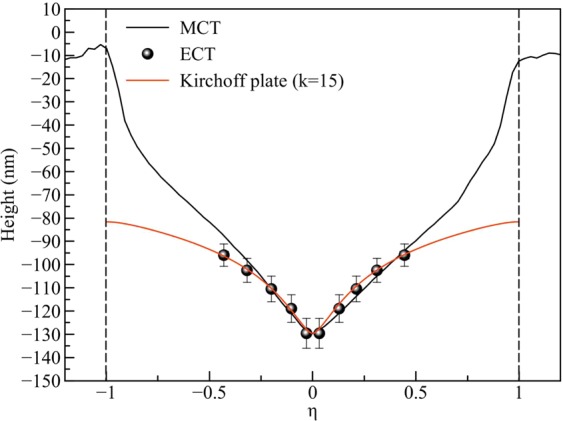
Comparison of the membrane deformations as obtained from the ECT and MCT measurements on the same membrane, and a comparison with the Kirchhoff plate model. Vertical dashed lines show the edge of membrane.

Similarly, the deformation of the membrane is revealed by the MCT measurement, where the STM tip is kept in contact with the membrane via its feedback loop while the AFM is scanned in contact mode. In this case, one would expect that also the STM is applying a force to the membrane^22^. However, it does not appear to be dominant in comparison to the contact mode AFM, as otherwise the tip should be visible in the AFM topography (like it is the case in tapping mode). Again, we assume that the deformation around the AFM tip moves along with the tip, in order to interpret the measured profile as the membrane shape around the tip. Both, the ECT measurement as shown in Fig. 7 and the MCT measurement in Fig. 9 were obtained on the same membrane, and are plotted for comparison in Fig. 12. The profiles agree within the experimental errors and agree with the Kirchhoff plate model for the central part of the deformation. The shape also qualitatively agrees with the calculated membrane shapes in^18^, however, in our case the suspended area and the deformation heights are much larger. Apparently, the membrane shape is given by the Kirchhoff model of a strained membrane only in the direct vicinity of the indentation by the AFM tip, while the shape further away from the tip might be dominated by other factors such as built-in slack from the preparation or the geometry of the support.

Finally, the appearance of the polymer contamination in the topography and MCT images as shown in Fig. 11 deserves further discussion. Based on the MCT image, the membrane and STM tip following its shape move up and down during scanning with AFM tip. When the AFM tip passes over the polymer, the STM tip moves downward by ~3 nm (Fig. 11d). This can not be an effect of only the sample topography: Any purely topographic feature on the membrane would be followed by the AFM z-feedback mechanism, and would leave the membrane position unchanged. Only if the force between the AFM tip and polymer contamination is different than the force between the tip and the cleaner regions of the MoS_2_ crystal, the membrane will change its position and this is revealed by the MCT measurement. In other words, the MCT measurement will show variations in the force between the AFM tip and the sample, but from its principle it can not be influenced by the topography. Hence, the MCT measurement provides a route to identify regions of different tip-sample interactions and thus different surface chemistry, in a way that is well separated from the topography. We thus expect that the MCT mode can complement AFM phase and friction imaging methods, which also reveal the chemistry of scanned surface^40–43^, in the case of free standing membranes.

## Conclusions

In this work, we showed the combination of AFM and STM measurements on free-standing 2D material membranes with the two probes accessing the membrane from opposite sides. We demonstrate novel imaging modes where a signal from one stationary tip is recorded as a function of the other (scanning) tip’s position. The local deformations induced by a scanning probe tip are measured using another scanning probe tip. From the electric and mechanical measurements we obtained the shape of the membrane around the indentation force from an AFM tip in contact mode. Both measurements agree with each other and the deformation close to the tip can be described by the Kirchhoff plate model of a strained membrane, while the larger scale of the membrane deviates from this model. Moreover, we demonstrated a new way to identify variations in interaction forces between one tip and the membrane in a way that is not influenced by the sample topology. We expect that the multi-probe SPM approach shown here will open new avenues towards exploring mechanical, chemical and electronic properties of ultra-thin membranes.

## Methods

### Sample preparation

The thickness of exfoliated flakes used in the experiments has been identified optically. Following the exfoliation and optical characterization, the samples were transferred on top of holey SiN/Si chips with the average pore size of 15 × 15 *μ*m, using a polymer-assisted transfer technique^17^. For the sample used in Fig. 11, we used a Quantifoil TEM grid made from a dissolvable plastic for transfer as described in the supplementary information. This transfer results in a residue pattern corresponding to the grid structure.

### Double-tip STM-AFM

The double-tip SPM housing probe scan AFM and STM scanners was built by DME (Danish Micro Engineering A/S, Herlev, Denmark, Supplementary Fig. 1). To carry out simultaneous AFM and STM measurements in double-tip SPM, the STM tip is first approached to the sample and usually lands on one of the metal-coated (Au(~10 nm)/Cr(~5 nm)) side walls of the pyramid being on the back side of the SiN/Si chip. Then, small lateral displacements or scans of the STM tip are used to measure the local gradient of the surface and thus the direction towards the center of the pyramid, which is followed by coarse displacement in the corresponding direction. After approaching the membrane from the back side by STM, the coarse adjustment of the AFM is done via an optical microscope image and the sample is scanned by the AFM. The coarse adjustment with the optical microscope is accurate enough to place the free-standing part of the membrane into the AFM scan area, and the STM tip can be found by its deformation in the membrane. AFM tips were purchased from NanoAndMore GmbH, Germany/NanoWorld, Switzerland. Mostly ARROW series AFM tips were used. The AFM probes have an arrow shape and their tip radius is smaller than 10 nm. The length, mean width and thickness of the cantilevers are in the range of 155–165 *μ*m, 40–45 *μ*m and 4.1–5.1 *μ*m, respectively (according to manufacturer’s specifications). AFM tips for electric measurements are coated by platinum-iridium (Pt/Ir). STM tips were self-made by electrochemical etching of tungsten wires^22^. The apex of STM tips is estimated to be ~26 nm by SEM measurements (see Supplementary Fig. 7). All experiments presented in this work have been carried out in air with no specific control of temperature and humidity; typical values for the temperature in the room were between 22 and 25 degrees Celsius and a relative humidity between 50 and 70 percent.

### Scanning and transmission electron microscopy

SEM and TEM images of the samples shown in Supplementary Information were recorded by a Delong instruments LVEM5 table-top transmission electron microscope operated at 5 kV.

## Supplementary information

Supplementary Information.
