# Supplementary material for: New imaging modes for analyzing suspended ultra-thin membranes by double-tip scanning probe microscopy
Source: Sci Rep. 2020 Mar 16;10:4839. doi: 10.1038/s41598-020-60731-x (PMC7076010; doi:10.1038/s41598-020-60731-x)
Supplement: Supplementary file 1 — Supplementary Information. [file 41598_2020_60731_MOESM1_ESM.pdf]

SUPPLEMENTARY INFORMATION
New imaging modes for analyzing suspended ultra-thin membranes by double-tip scanning probe microscopy

Kenan Elibol[1,*], Stefan Hummel[1,*], Bernhard C. Bayer[1,2], and Jannik C. Meyer[1,3,†]

[1]*Faculty of Physics, University of Vienna, Boltzmanngasse 5, A-1090, Vienna, Austria*

[2]*Institute of Materials Chemistry, Vienna University of Technology (TU Wien), Getreidemarkt 9/165, A-1060 Vienna, Austria*

[3]*Institute for Applied Physics, University of Tübingen, Auf der Morgenstelle 10, 72076 Tübingen*

[*]*These two authors contributed equally to the work*

[†]*Corresponding author; E-mail: jannik.meyer@uni-tuebingen.de*

Double-tip SPM. AFM and STM scanner housed in double-tip SPM are shown in Supplementary Figure 1. Both tip-scan scanners can be operated independently while the sample is stationary. Additional schematics of the sample geometry and an scanning electron microscopy (SEM) and transmission electron microscopy (TEM) image of the sample is shown in Supplementary Figure 2. The TEM image in Figure 2c also shows the polymer residue resulting from a transfer using the plastic quantifoil grids as for the experiment in Figure 11 of the main manuscript.

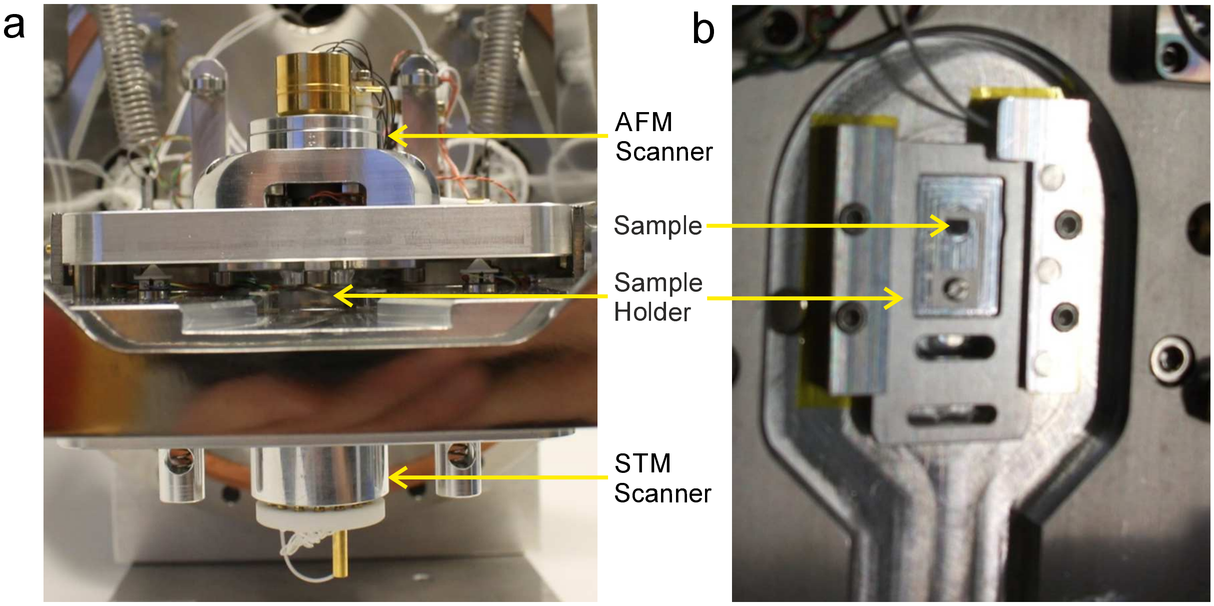

Supplementary Figure 1: (a) A photograph showing the AFM and STM scanners in DXSPM. (b) A photograph of the stage including sample holder with a SiN/Si chip mounted.

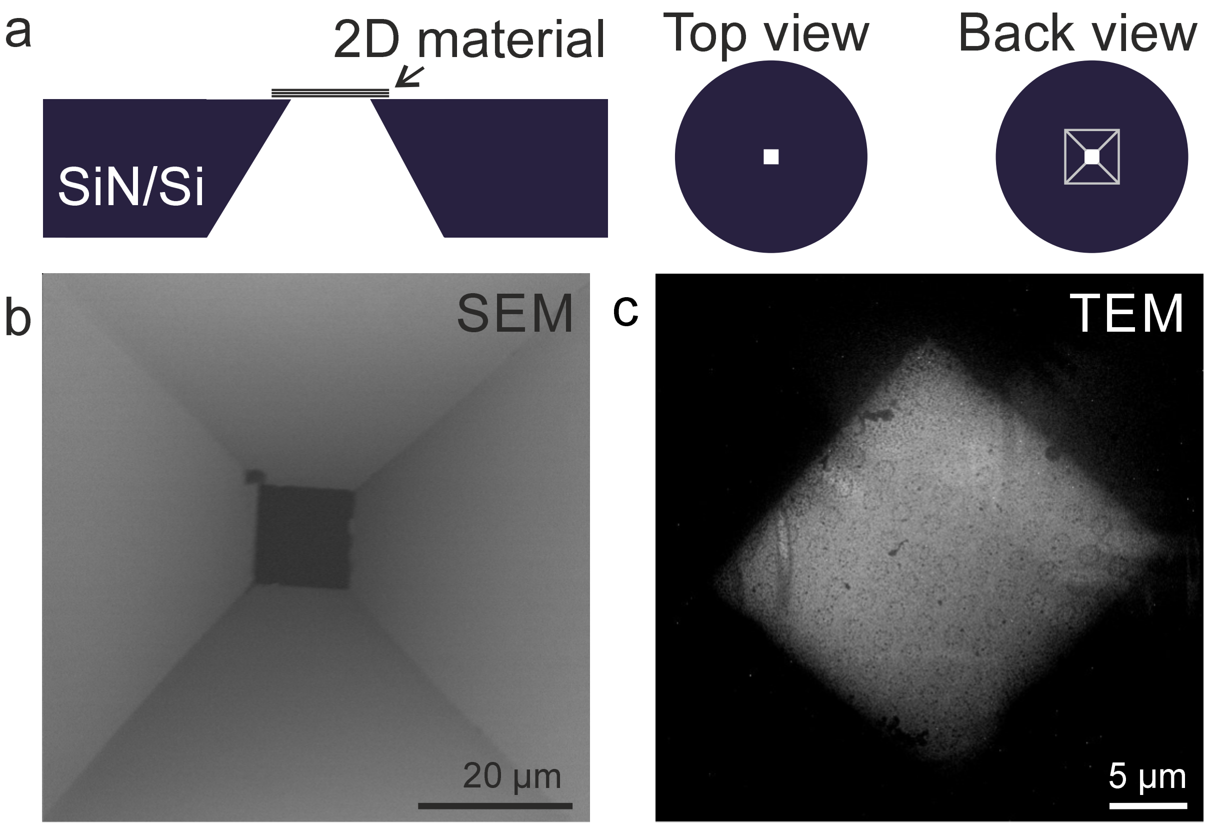

Supplementary Figure 2: (a) Schematic illustration of a sample on a SiN/Si chip. (b) SEM image of the back side of the SiN/Si chip. (c) TEM image showing the electron transparent areas of this chip.

Supplementary Figure 3 shows the individual AFM and STM topography images of a few-layer graphene membrane. The circular patterns appearing at the topography image are polymer residues, which are sticking to the top side of the sample after it is transfered using a TEM grid with a plastic support film [17]. Therefore, the opposite side of the flake, scanned by STM tip, should not contain any polymer residue (and certainly it can not contain residue in a pattern). The Si(111) corners of the pyramid shaped hole at the back side of SiN/Si chip are shown in the STM topography image in panel b. To demonstrate the capability of the STM, an atomic resolution image of a HOPG crystal is shown in panel c. This atomic resolution STM image of the graphite show a hexagonal lattice with the expected periodicity of 0.246 nm. The inset shows a Fourier transform of the image.

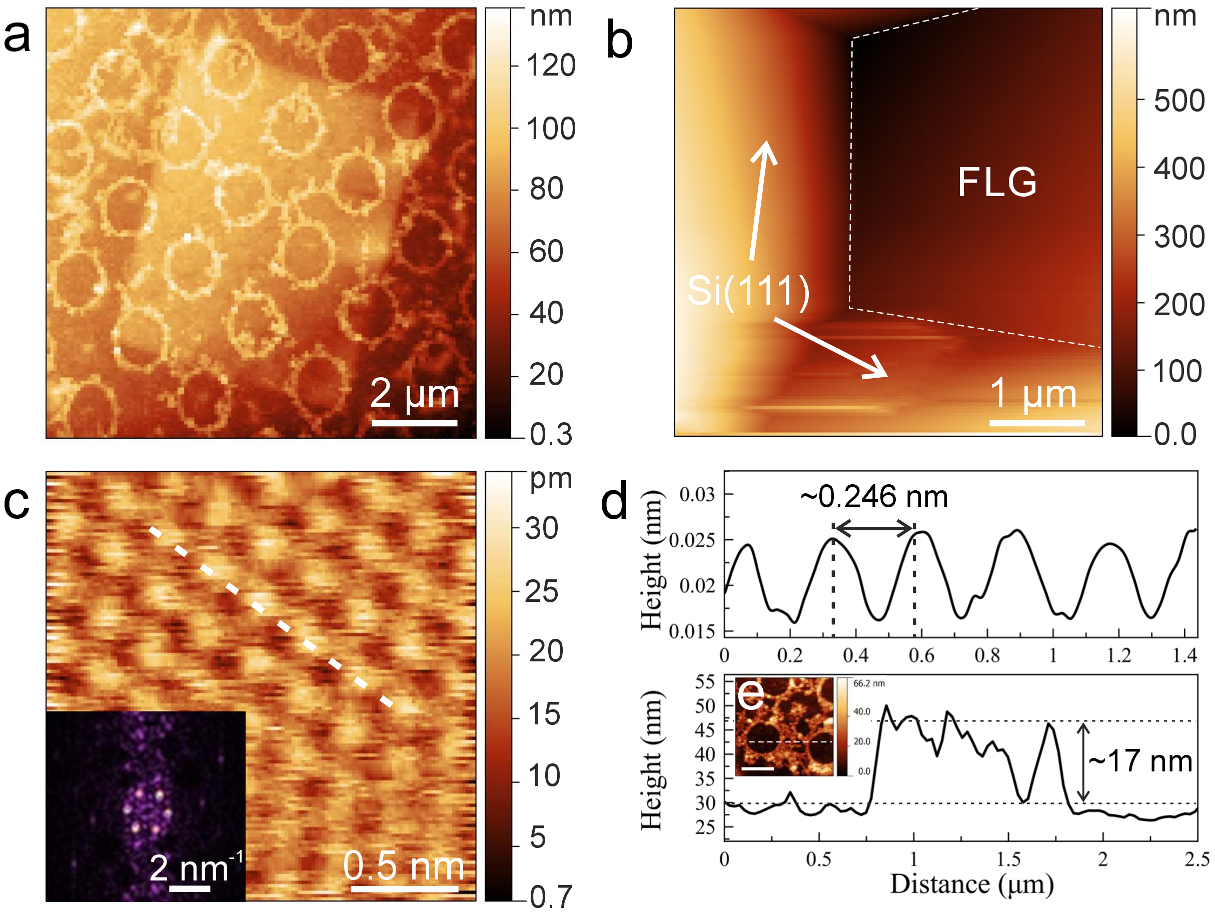

Supplementary Figure 3: (a) AFM and (b) STM topography of the suspended few-layer graphene on a SiN/Si chip with $\sim$3 μm $\times$ 4 μm frame. (c) The atomic resolution STM image of a highly oriented pyrolytic graphite (HOPG). Tunneling parameters for the STM tip are (a) U= 0.1 V, I= 0.4 nA and the inset shows a Fourier transform. (d) Height profile along the white dashed line on the atomic resolution STM image. (e) Height profile along the white dashed line on the AFM image shown as inset (similar as in a), showing the height of the polymer contamination. The scale bar in the inset of panel e is 1 μm.

Modelling membrane's deflection by Kirchhoff plate theory (Ref. [28]). The deformation behavior of graphene under point load is shown in Supplementary Figure 4a for different values of in-plane tension. The membrane behavior is observed for the in-plane tension values larger than 1. In Supplementary Figure 4b, calculated normalized deflection is shown for different k values. In the calculations, we ignore that the membrane is not circular.

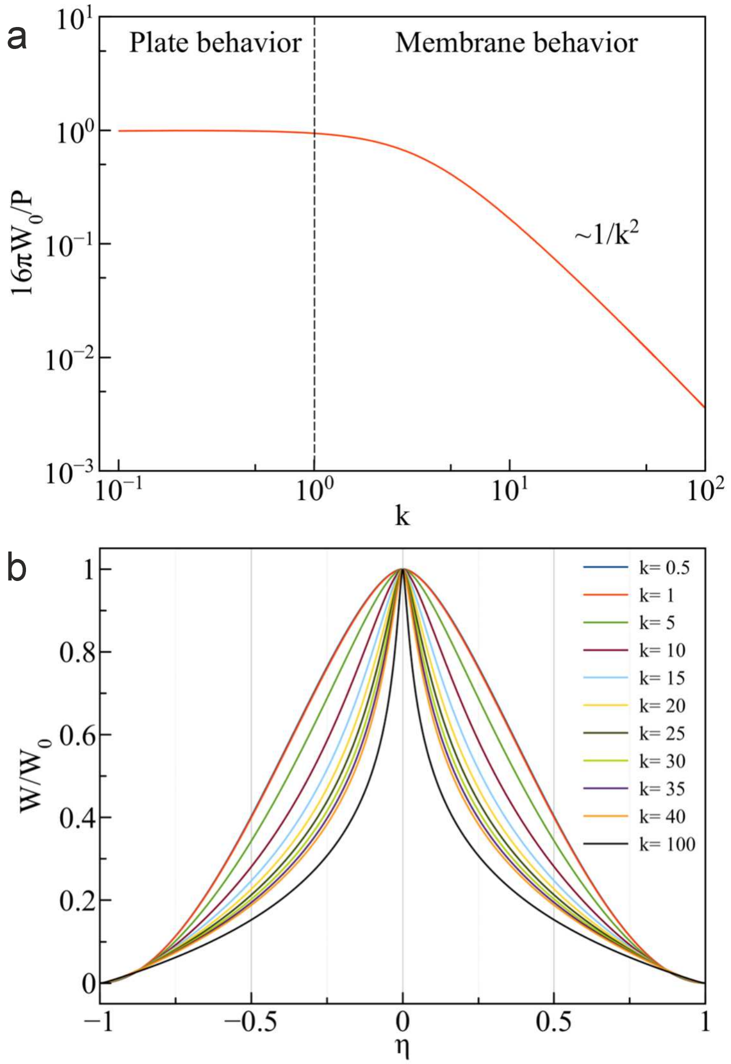

Supplementary Figure 4: (a) Central deflection normalized by non-dimensional loading P versus in-plane tension k. (b) Normalized deflection versus normalized radial distance η for different values of in-plane tension k.

MCT recorded at different tunneling parameters. Topography and MCT images of the MoS_2 membrane shown in Figure 10 are presented for different tunneling parameters in Supplementary Figure 5. In MCT images, 1.3 V corresponds to change in the position of STM z-piezo by $\sim$50 nm.

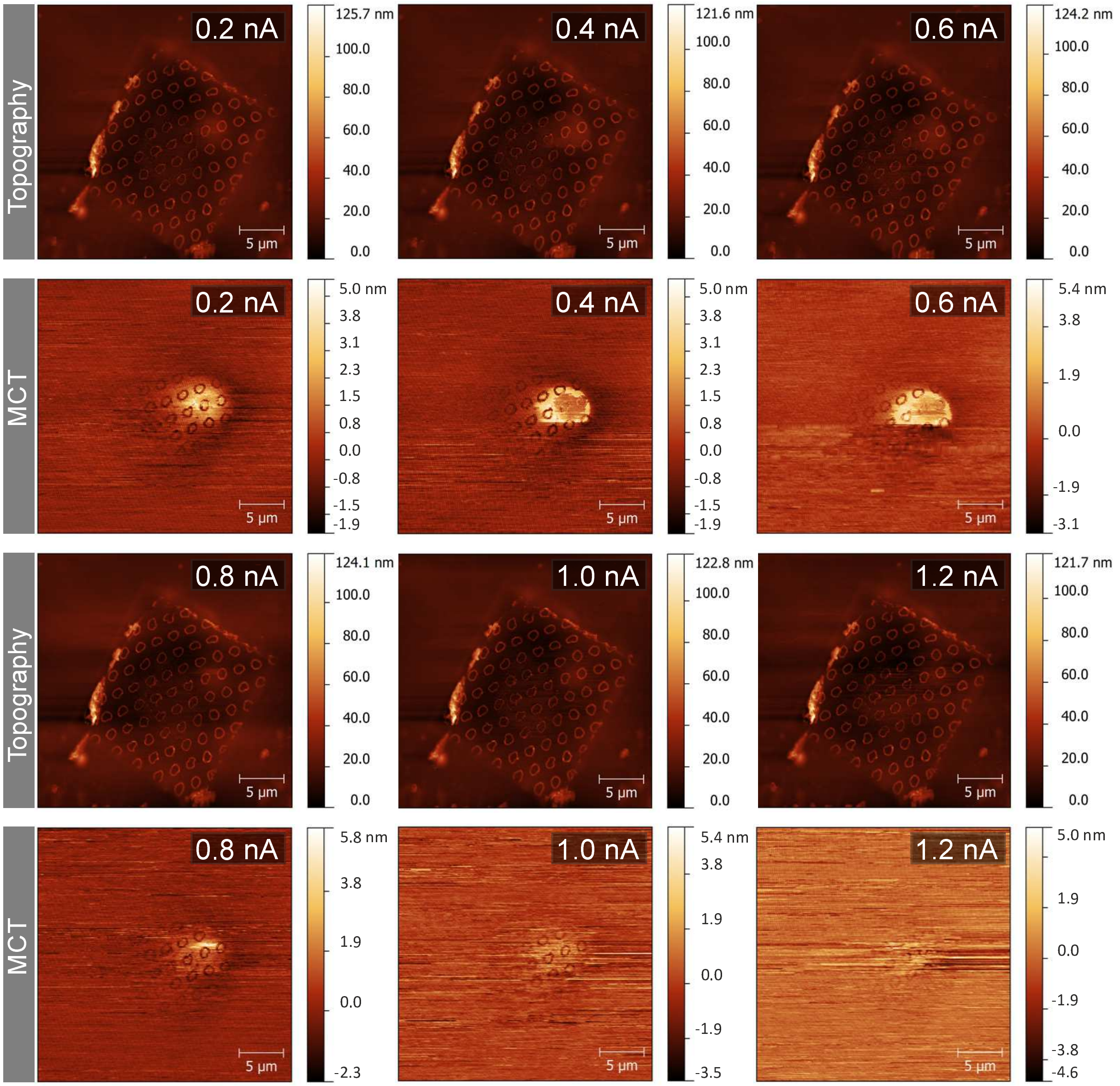

Supplementary Figure 5: Topography and MCT images of a multilayer MoS_2 membrane for different STM tunneling current values. The bias voltage is 1V in all scans.

Determination of strain. The strain applied on a membrane by a tip is estimated by an analytical model (see equation 2) [44]. Here, the total strain by a point deformation is the sum of radial and circumferential components (see Supplementary Figure 6). The force applied by a tip and the sum of strain on a membrane is given by

$$F = E(q^3 ah)\left[\frac{\delta}{a}\right]^3 \tag{1}$$

$$\varepsilon_{total} = \left[\frac{F^2}{3\pi^2 E^2 h^2}\right]^{\frac{1}{3}}\left[\frac{1}{r}\right]^{\frac{2}{3}} \tag{2}$$

where h is the thickness of the graphene, δ is indentation depth, E is Young's modulus, F is force applied by the tip to membrane, ν is Poisson ratio (0.165), and $q = 1/(1.05 - 0.15\nu - 0.16\nu^2)$. For the graphene, E = 1 TPa, ν= 0.165 and q= 0.97. The r is the radial distance to the center of deformation created by a tip. In supplementary figure 6, the strain is plotted from the tip radius (25nm) up to the membrane diameter. Even directly at the tip, it is less than 0.05% according to the analytical model.

These values are consistent with our previous double tip STM/STM experiments [22], where strain levels from similarly STM tip induced deformations of few layer graphene membranes were estimated to reach a maximum of only ∼0.1%. They are also consistent with previous experiments which employed coupled contact mode AFM nanoindentation with laterally resolved, optical Raman mapping of strain levels in suspended few layer graphene membranes indicated that strain levels reach a maximum of only ∼0.5% directly under the tip even for high AFM tip indentation forces [17]. Combined, this corroborates that significant changes in electronic properties of our membranes from our currently small tip induced strain levels are unlikely.

It has been theoretically shown that the uniaxial strains below ∼20% cannot open a band gap in graphene [45]. In an experimental work, it is also shown that no band gap opening is observed for graphene under moderate uniform strain of up to 3% [46]. Further, semiconductor-metal transition of MoS_2 occurs at high levels of tensile strain (10% for monolayer MoS_2 and 6% for bilayer MoS_2) [35]. In our experiments, the strain estimated is less than 0.05%, and hence the change in the electronic properties of suspended graphene and MoS_2 is not expected to be significant.

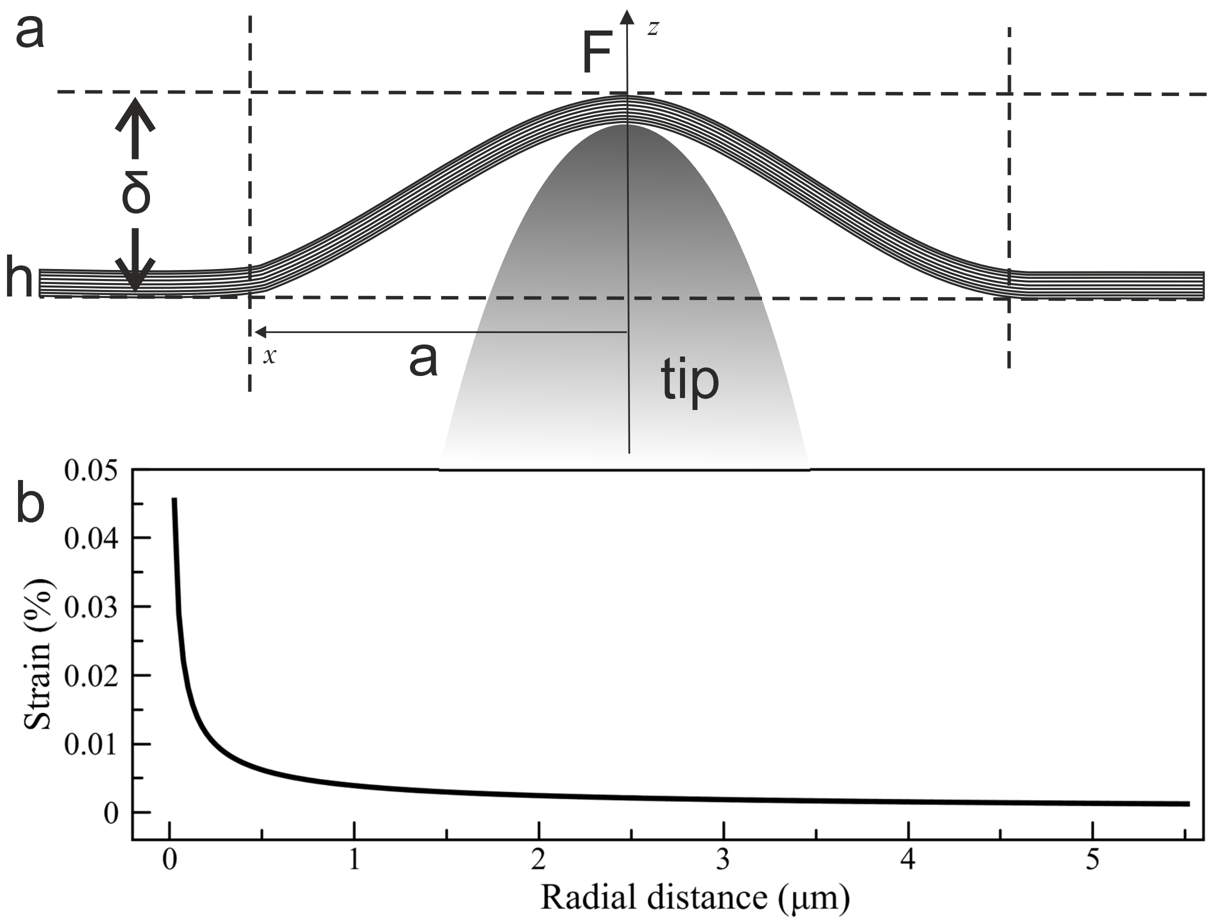

Supplementary Figure 6: (a) Schematic of a strained graphene membrane. (b) Estimated strain applied on suspended multilayer graphene by the tip. The δ and a are 38.52 nm and 6.25 μm, respectively.

Estimation of STM tip radius.

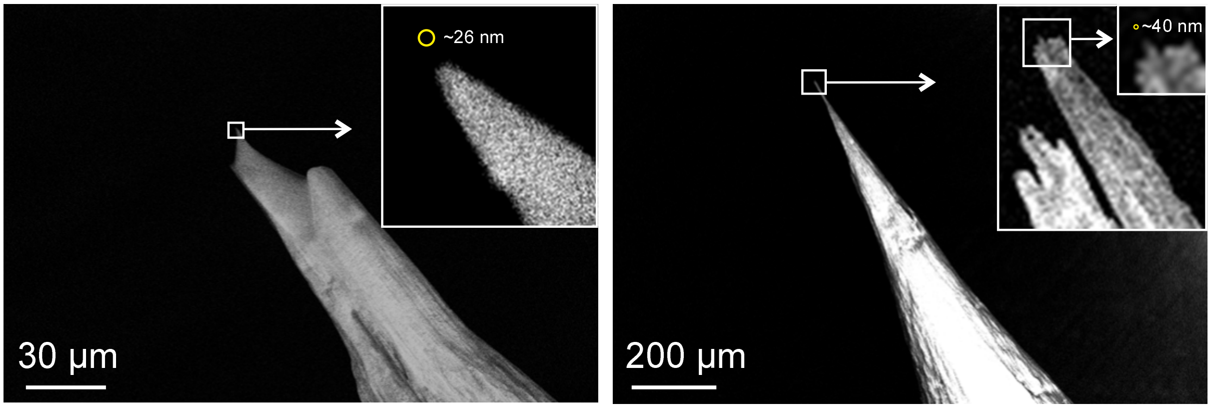

Supplementary Figure 7: SEM images of two different STM tips. Based on SEM images, the radius of STM tips are estimated to be ∼26 nm. In the previous work of Eder *et. al* [22], the apex of STM tips prepared by the same method was estimated to be ∼20 nm by TEM imaging. The yellow colored circles with a radii of ∼26 nm and ∼40 nm are for comparison.

References

[44] Ryan Beams, Luiz Gustavo Cancado, Ado Jorio, A Nick Vamivakas, and Lukas Novotny. Tip-enhanced raman mapping of local strain in graphene. *Nanotechnology* 26(17): 175702, 2015.

[45] Vitor M. Pereira, A. H. Castro Neto, and N. M. R. Peres. Tight-binding approach to uniaxial strain in graphene. *Phys. Rev. B* 80:045401, 2009.

[46] Mingyuan Huang, Tod A. Pascal, Hyungjun Kim, William A. Goddard, and Julia R. Greer. Electronicmechanical coupling in graphene from in situ nanoindentation experiments and multiscale atomistic simulations. *Nano Letters* 11(3):12411246, 2011.
